# Nuclear environmental DNA resolves fine-scale population genetic structure in an aquatic habitat

**DOI:** 10.1016/j.isci.2023.108669

**Published:** 2023-12-07

**Authors:** Zifang Liu, Mary A. Kishe, Nestory P. Gabagambi, Asilatu H. Shechonge, Benjamin P. Ngatunga, Katie Smith, Andrew D. Saxon, Alan G. Hudson, Tyler Linderoth, George F. Turner, Rupert A. Collins, Martin J. Genner

**Affiliations:** 1 School of Biological Sciences, University of Bristol, Life Sciences Building, 24 Tyndall Avenue, Bristol BS81TQ, UK; 2 Tanzania Fisheries Research Institute (TAFIRI), P.O. Box 9750, Dar es Salaam, Tanzania; 3 Department of Genetics, University of Cambridge, Cambridge CB2 3EH, UK; 4 W.K. Kellogg Biological Station, Michigan State University, Hickory Corners, MI 49060, USA; 5 School of Biological Sciences, Bangor University, Bangor, Gwynedd LL57 2UW, UK; 6 Department of Life Sciences, The Natural History Museum, Cromwell Road, London SW7 5BD, UK

**Keywords:** Environmental science, Genetics, Techniques in genetics, Evolutionary biology

## Abstract

There is considerable potential for nuclear genomic material in environmental DNA (eDNA) to inform us of population genetic structure within aquatic species. We tested if nuclear allelic composition data sourced from eDNA can resolve fine scale spatial genetic structure of the cichlid fish *Astatotilapia calliptera* in Lake Masoko, Tanzania. In this ∼35 m deep crater lake the species is diverging into two genetically distinguishable ecomorphs, separated by a thermo-oxycline at ∼15 m that divides biologically distinct water masses. We quantified population genetic structure along a depth transect using single nucleotide polymorphisms (SNPs) derived from genome sequencing of 530 individuals. This population genetic structure was reflected in a focal set of SNPs that were also reliably amplified from eDNA — with allele frequencies derived from eDNA reflecting those of fish within each depth zone. Thus, by targeting known genetic variation between populations within aquatic eDNA, we measured genetic structure within the focal species.

## Highlights

• Intraspecific genetic variation can be identified within aquatic environmental DNA
• Allele frequencies in eDNA match those in local source populations
• Environmental DNA can be used to quantify population genetic divergence

## Graphical abstract

**Figure:**
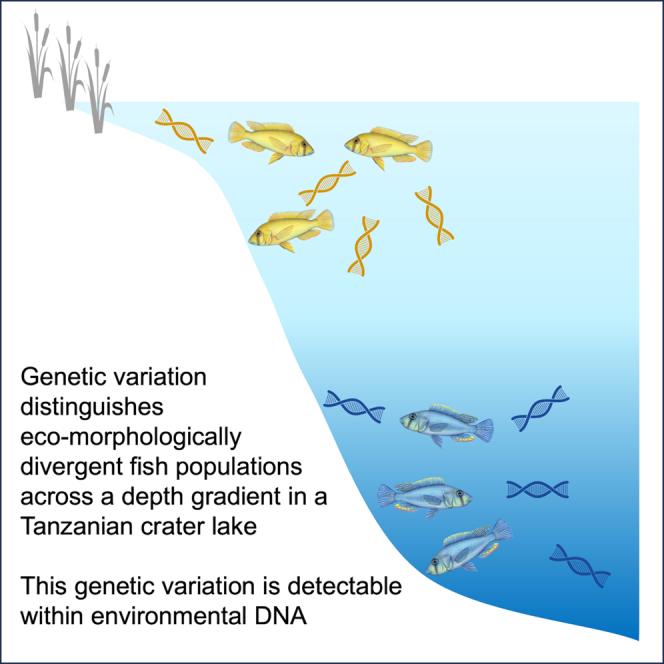

## Introduction

There is growing interest in the use of aquatic eDNA for studying population genetics of focal species.^1^^,^^2^^,^^3^ To date much of this research has focused on variation in target regions of the mitochondrial genome, and it has been possible to resolve spatial genetic structure in a range of vertebrate species.^2^^,^^3^^,^^4^^,^^5^ Mitochondrial DNA (mtDNA) has been employed for population genetic inference in this context as it is reliably amplified from eDNA and sequenced, and the relatively high evolutionary rate allows populations to be distinguished. Moreover, since variable sections of mtDNA are often flanked by phylogenetically conserved regions, useful primers can be readily identified, and eDNA-derived sequences can be assigned to focal species.^6^ However, mtDNA may be suboptimal for resolving genetic structure among very recently diverged populations; it is typically maternally inherited and non-recombining, so the whole mtDNA genome acts as a single locus and primarily provides only information on female ancestry.^7^ Furthermore, nuclear insertions of mitochondrial origin (numts), or selection on the mitochondrial genome, can also confound population genetic inference.^7^^,^^8^ Thus, there has been interest in utilizing nuclear eDNA for population genetic analysis.^3^^,^^9^^,^^10^^,^^11^^,^^12^ To date, support for nuclear eDNA as a reliable tool for population genetic inference is limited, although it has been shown that microsatellite allele frequencies of round goby (*Neogobius melanostomus*) derived from eDNA have a strong correspondence to allele frequencies of source fish, in both mesocosms and the natural environment.^11^ Importantly, it has also been shown that eDNA-derived microsatellite allele frequencies of round goby can be used to describe structure among geographically separated populations, and that this pattern closely resembles that derived from microsatellite-based analyses of tissue samples.^12^ Here we build on this research by investigating if population genetic structure can be determined over a small spatial scale within a single lake using single nucleotide polymorphism (SNP) variants from the nuclear genome.

## Results

**Figure 1:**
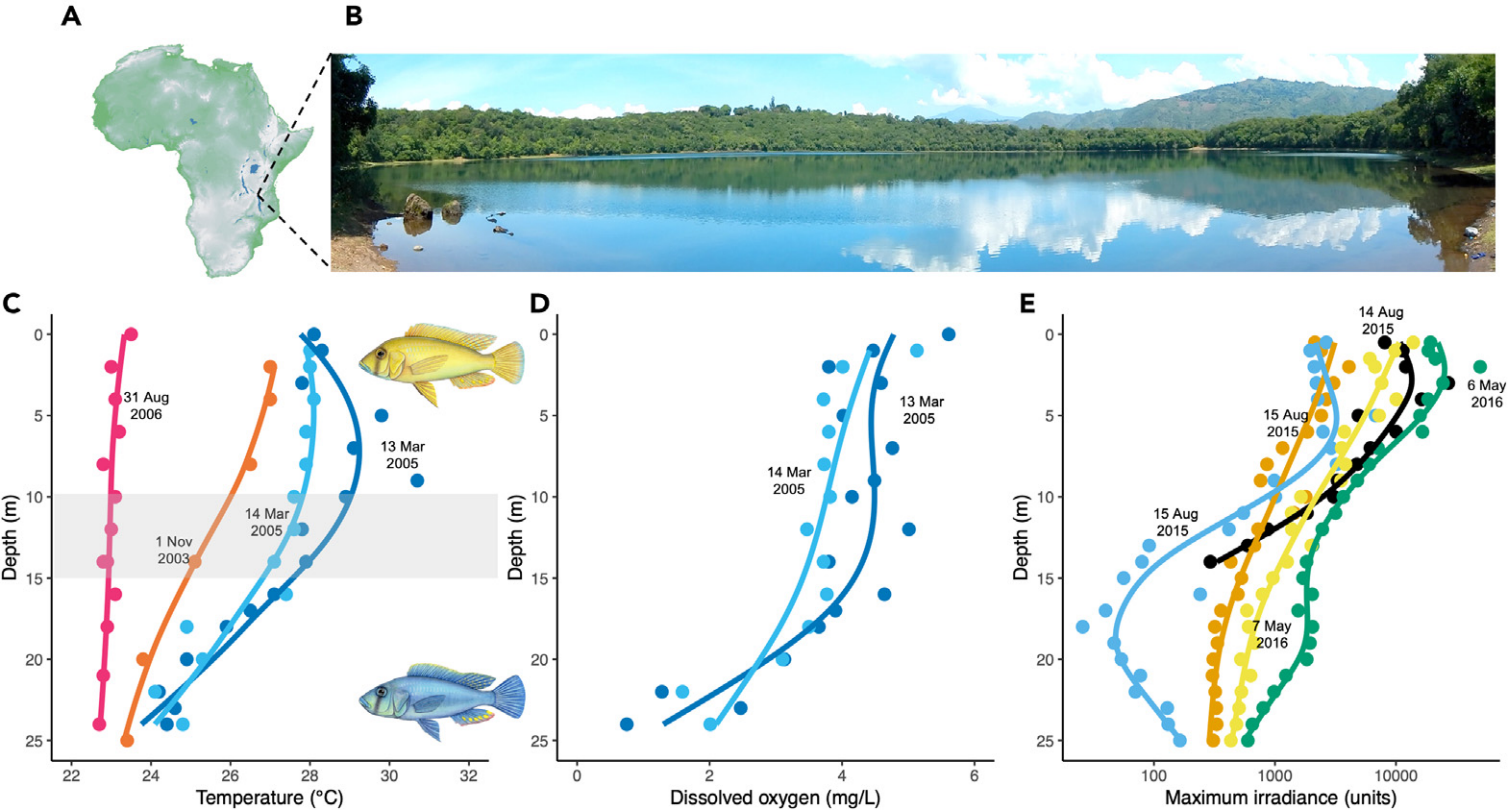
The study location (A and B) Lake Masoko crater lake in southern Tanzania. (C–E) The lake has a vertical gradient of decreasing temperature, dissolved oxygen and light levels with increasing depth, with differences most prominent either side of a thermo-oxycline at 10–15 m. The littoral ecomorph with yellow breeding males dominates the shallower waters 0–10 m, while the benthic ecomorph with blue breeding males dominates the deeper waters 15–25 m. Separate lines denote different sampling dates. Each point represents one measurement. Temperature and oxygen data are from ref.^13^. Sidewelling light data were collected using an Ocean Optics USB2000+VIS-NIR-ES spectrometer with a cosine corrector, measuring 350–1000 nm, with a 40 m fiber optic cable.

Our study system is Lake Masoko, a crater (maar) lake in southern Tanzania that has no surface connections with nearby rivers^13^^,^^14^ (Figures 1A and 1B). The lake is ∼700 m in diameter, has a maximum depth of ∼35 m with rapid attenuation of light with increasing depth (Figure 1E). The lake exhibits seasonal stratification, with a thermo-oxycline located at approximately 15 m (Figures 1C and 1D). It has been proposed that there is stronger stratification during the warmer wetter season (approximately September–March) compared to the cooler and drier season (approximately April–August),^15^ although the consistency of this pattern across years is unknown.

**Figure 2:**
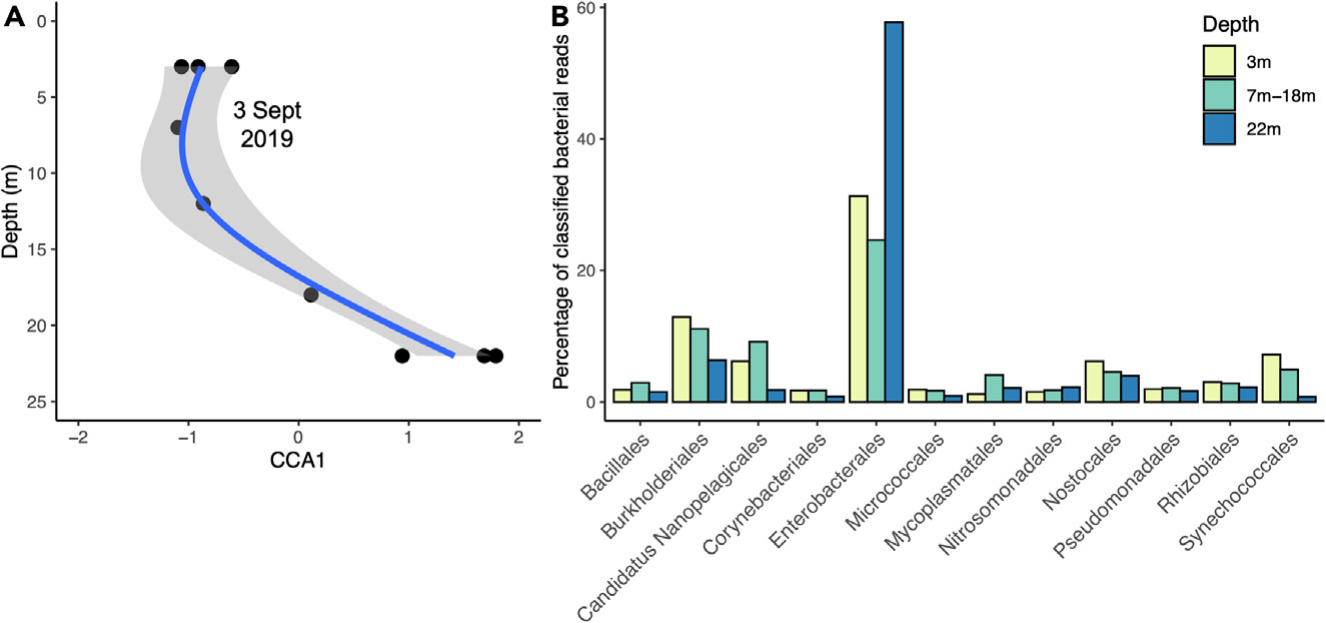
The microbial community of Lake Masoko (A) The primary axis of microbial community composition (CCA axis 1) shows community change with increasing depth. The line is fitted with a generalized additive model and the shaded area indicates the 95% confidence interval. (B) The average proportion of reads assigned to microbial orders within surface (3 m, n = 3), mid-depth (7m–18 m, n = 3) and deep (22 m, n = 3) waters of the lake.

We collected environmental DNA using SCUBA from five depths in September 2019 (3, 7, 12, 18 and 22 m). To confirm two ecologically distinct water masses are present, we used Oxford Nanopore Technologies (ONT) sequencing on nine samples of bulk environmental DNA (Table S1). We mapped the derived reads to a microbial database (minikraken_20171019_8GB; https://ccb.jhu.edu/software/kraken) and quantified the number of reads assigned to each microbial order. We found a significant association between the composition of the microbial community and increasing depth (Canonical Correspondence Analysis, Anova *F*_1,7_ = 4.582, p = 0.009), with the major axis of variation (CCA1) capturing a switch in community composition between 10 and 20 m (Figure 2A), coincidental with the known position of the thermo-oxycline at approximately 15 m (Figures 1C–1E). This shift in the functional composition of the microbial community reflected the change in environment, with shallow, well-oxygenated, warmer and more brightly lit waters possessing a higher proportion of photosynthetic cyanobacteria (e.g., Synechococcales), and deeper, poorly oxygenated, cooler and darker waters possessing a greater dominance of anaerobic bacteria (e.g., Enterobacterales) (Figure 2B).

The lake contains a pair of ecomorphs of the cichlid fish *Astatotilapia calliptera*. A littoral ecomorph with yellow males dominates the shallow waters (<5 m) and feeds primarily on benthic invertebrates, while a deep ecomorph with blue males dominates the deeper waters (>20 m) and feeds primarily on zooplankton.^14^^,^^16^^,^^17^ The ecomorphs show strong differences in ecologically relevant morphologies, including body shape and lower pharyngeal jaws – which are used principally for processing prey.^14^^,^^16^ Using whole genome sequence (WGS) data of individuals collected from known depth strata, the ecomorphs have been shown to be genetically divergent,^18^ and these genomic data show the prominent shift in the relative abundance of ecomorphs over the depth gradient,^18^ although the total abundance of each ecomorph at each depth is not known. Using our eDNA from the five depths (3, 7, 12, 18, and 22 m), we tested if we could identify genomic DNA from the focal species in the environmental DNA. We also tested whether it was possible to determine differences in allele frequencies at SNP loci that had previously been inferred to be segregating between ecomorphs along the depth gradient.^14^

We focused our analyses on a subset of 120 SNPs which had previously been identified within a set of 98 loci across the *A. calliptera* genome.^14^ To amplify loci containing our target SNPs, we used PCR on 16 eDNA samples (Table S2), with an average amplicon length of 84.86 bp (range: 49–109 bp). The resulting amplicons were sequenced using an Illumina NextSeq 550. The generated sequences were mapped to the *A. calliptera* genome (fAstCal1.2), with an average mapping success of 52.56% of reads (range 14.70–97.89%; Table S3). The mapped reads were filtered to include only target regions, and within them we located 114 of the 120 SNP positions in the eDNA read data. We used read counts to quantify variation (allele frequencies) in each of the focal SNPs. Of these 114 SNPs, 102 were variable in the eDNA samples. We further filtered the SNPs to only include those present in 12 or more of the 16 samples. This resulted in a set of 71 SNPs, that we refer to here as “eSNPs”.

**Figure 3:**
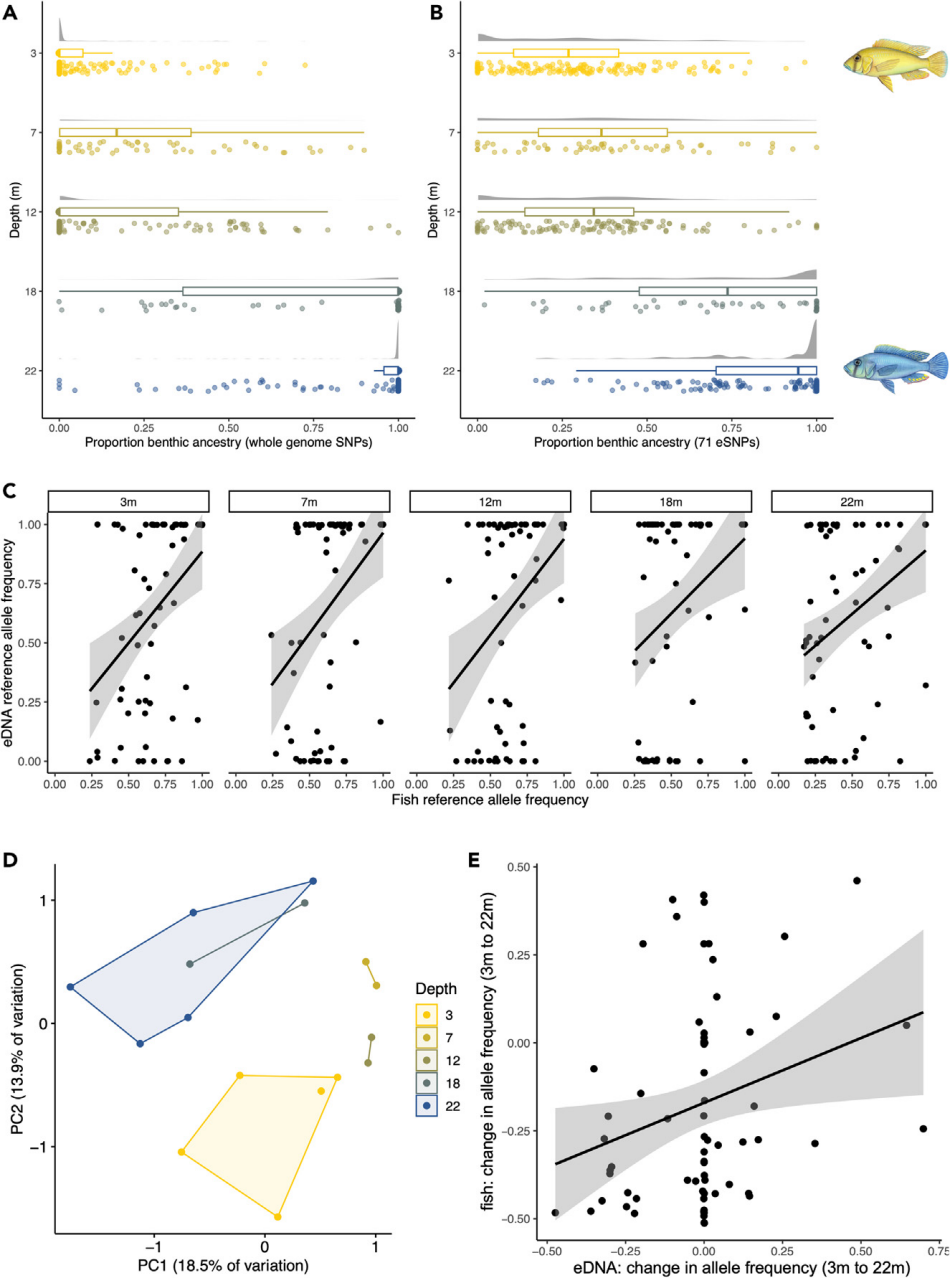
Population genetic structure inferred from DNA sourced from tissue and eDNA Results of admixture analyses of fish tissue genome samples assuming two ancestry components (K = 2) using data from (A) the whole genome (773,357 SNPs after filtering for linkage disequilibrium) and (B) 71 focal eSNPs. Each datapoint represents one individual fish, and the same 530 fish are present in both analyses. (C) Correspondence between allele frequencies in fish (WGS) sampled from each depth stratum, and mean allele frequencies from eDNA sampled from the same stratum. Each point is one eSNP. The line is fitted with a linear model, and shading represents the 95% confidence interval. (D) PCA of eDNA allele frequencies in each sample using the eSNPs. Each point represents one eDNA sample. (E) Correspondence between shifts in mean reference allele frequencies in eDNA samples from 3 to 22 m and shifts in reference allele frequencies in fish (WGS) sampled between 3 and 22 m, for the 71 eSNPs. The line depicts the fit of a linear model, and shading represents the 95% confidence interval.

We tested if these eSNPs can resolve population genetic structure over the depth gradient in fish samples collected from known depths between 2013 and 2018, that have had their whole genomes sequenced (n = 530 individuals^18^; 3,881,258 biallelic SNPs). Individual ecomorph ancestry estimates using 773,357 unlinked SNPs showed a clear break in population genetic structure between 12 m and 18 m, coincident with the location of the thermo-oxycline (Figure 3A). Using the 71 eSNPs, it was possible to resolve a similar pattern across fish from the five depth strata, although with a less polarized assignment of individuals to the two population genetic clusters (Figure 3B).

If allelic compositions of known SNPs in fish were reflected in eDNA, we expected a positive association between the frequency of the reference allele (i.e., the allele matching the reference genome) in the fish from the whole genome data, and the frequency of the same allele in the eDNA, within each depth band. Focusing on the eSNPs, at each depth, there was a significant positive association between the reference allele frequencies between the eDNA and WGS datasets (Figure 3C; Table 1), confirming the non-random nature of the allelic composition of eDNA samples.

**Table 1:** Tests of association between *A calliptera* reference allele frequencies in eDNA and WGS data across eSNPs (Pearson’s correlations)

Depth (m)	*r*	*df*	*t*	*P*
3	0.403	69	3.716	0.00041
7	0.404	68	3.639	0.00052
12	0.386	69	3.483	0.00087
18	0.344	68	3.024	0.00352
22	0.385	69	3.470	0.00090

A Principal Component Analysis (PCA) of the eDNA genetic variation across the eSNPs revealed clear differences between the deepest and shallowest sample groupings (Figure 3D). To determine whether genetic variation among the ecomorphs corresponded to the variation among the eDNA eSNPs, we compared the change in WGS and eDNA eSNP reference allele frequencies for fish sampled from 0 to 5 m to those sampled at 20-25 m. Overall, we found a significant positive association between changes in the allele frequencies in the eDNA, and the changes in allele frequencies of the fish (Pearson’s correlation, n = 71, *r*^2^ = 0.268, p = 0.0237; Figure 3E).

## Discussion

Our study demonstrates that the clear depth differences in genetic structure of the Lake Masoko *A. calliptera* ecomorphs are reflected in eDNA present in their immediate environment. This pattern is not consistent with complete homogenization of DNA in the water column, although we consider it plausible that there is transport of cichlid fish eDNA between the depth zones via passive mechanisms (for example, fecal particles) or active processes (for example the movement of their predators). Such processes may partially explain the variation in the association between allele frequencies observed in sampled fish (WGS) and the allele frequencies observed in the eDNA.

It seems most likely that the differences in eDNA-based allele frequencies are due to strong associations of *A. calliptera* individuals to specific shallow or deep-water habitats, with little migration between depths – at least during the sampling period when there was stratification of the water column. Although the movements of these fish have not been individually tracked, there is clear support for habitat-specific adaptation in adults, from phenotypic,^14^^,^^16^^,^^17^ genomic^14^^,^^16^^,^^18^ and transcriptomic^16^^,^^17^ evidence. Environmental regimes, including light, oxygen and food resources have plausibly led selection to favor philopatry, with strong negative effects on fitness for individuals with inappropriate phenotypes for specific habitats.^19^ Notably, work to date showing strong genetic and phenotypic differences between the ecomorphs has been focused on adult individuals, but the divergence in eDNA allele frequencies between the depth zones either side of the thermo-oxycline is consistent with philopatry at all life history stages. The focal species *A. calliptera* is a maternal mouthbrooder,^20^ and also exhibits a short period of parental care after first release of free-swimming fry, so there is scope for habitat imprinting that may contribute to strong life-long habitat affiliation. Whether the eDNA we detected is disproportionately contributed by adults, subadults or juveniles is, however, unclear. While larger individual adults can generate eDNA at a greater rate than individual juveniles,^21^ contributions to total eDNA by each life stage will depend on their relative abundance, biomass and activity levels.^22^

Evidence of spatial structuring of allele frequencies within eDNA across the small spatial scale of less than 20 m clearly confirms the potential for eDNA to be used to describe fine scale spatial population structure in aquatic species. Studies in both freshwater and marine systems have also revealed the potential for fine scale differences in environmental DNA composition over narrow depth gradients, but those studies have focused on community structure. For example, in shallow Canadian lakes (13–30 m deep), there was clear differentiation in the fish species resolved between depth intervals when the lakes were thermally stratified – matching the distribution of the fish species – however, the differences in community structure among depth intervals were not present when the lakes were mixed.^23^ Similarly, in a New Zealand fiord, stratification in a halocline led to differentiation in fish, crustacean and echinoderm community composition resolved using eDNA over a depth only 4 m.^24^ Even in the absence of a clear water mass boundary differences fish community structure have been resolved over a depth gradient of only 10 m (Californian kelp forests^25^). It is becoming clear therefore that eDNA may be useful to describe not only gradients in diversity across larger spatial scales as is commonly recognised,^26^ but also over fine spatial scales in some systems, such as Lake Masoko, that are characterized by limited water movement and/or stratification.

Our study shows that sufficient information can be gained from nuclear SNP allelic frequencies from environmental DNA to infer population genetic structure. Studies that have explored the capacity for population genetics using eDNA data until now have focused on mtDNA,^4^ sequence capture by hybridization^9^ or nuclear microsatellites.^11^^,^^12^ We were able to benefit from *a-priori* knowledge of segregating SNPs in our focal species within a well characterized environment. Targeting SNPs dispersed throughout the nuclear genome in this manner has an advantage over shotgun sequencing eDNA as it enables taxonomic specificity – but may suffer from PCR biases. Use of DNA hybridization-capture techniques to select target regions of interest from eDNA, as has been applied to amplifying species-specific mitochondrial^27^^,^^28^ and nuclear^9^ regions from eDNA, may enable us to overcome implicit PCR biases.

Reference and re-sequenced genomes enable identification of SNPs for target species suitable for eDNA-based population genetics.^29^ More widespread availability of genomes from non-target species in communities would help to confirm the source species of sequenced fragments. In Lake Masoko, the dominant fish species is *Astatotilapia calliptera*, but three other fish species are present,^30^ including the cichlid *Coptodon rendalli* (estimated to have diverged from *A. calliptera* 18 Mya^31^), the cichlid *Oreochromis squamipinnis* (estimated to have diverged from *A. calliptera* 23 Mya^31^), the catfish *Clarias gariepinus* (estimated to have diverged from *A. calliptera* 224 Mya^31^). In addition, there are many domestic and wild terrestrial vertebrates in the Lake Masoko crater generating environmental DNA that can enter the water. It is possible that primers would also amplify fragments of eDNA from these or other sources, which may vary spatially in the lake, and this may explain the high variation in mapping success (samples ranged from 14.7 to 89.5%; Table S3). We focused on known biallelic SNPs with previously characterized allelic variation, and this will have reduced the influence of heterospecifics on our results. Implementation of data filtering steps that remove reads that map more closely to heterospecifics in the environment would be additionally beneficial, and this process would ideally benefit from a bespoke array of heterospecific reference sequences for the study site.

In principle, population-genomic methods using aquatic environmental DNA could become important for studying species that are challenging to sample due to rarity, catchability or habitat occupancy,^1^ or because their capture and direct sampling may be ethically questionable.^32^ This study has contributed to this field of research by confirming that nuclear loci can be reliably amplified from environmental DNA, and that it is possible to generate allele frequencies that can be used for population genetic inference. To conclude, our results strengthen indications that future research aimed at refining eDNA-based population genomic methods may improve our understanding of population structure of many species, including those of commercial, ecological and conservation importance, and within marine, estuarine and freshwater systems.

### Limitations of the study

Certainly, the results will be influenced by a multitude of factors, including the eDNA collection, preservation, extraction, PCR, sequencing and bioinformatic methods. We may expect PCR to distort allele frequencies, and the approach we used did not enable us to check for consistency across PCR replicates. There are also likely to be seasonal differences in the ability to resolve spatial population genetic structure, related to the strength and distribution of stratification, and breeding seasonality may also influence the density of eDNA in the water^33^^,^^34^ (observational evidence indicates our focal species *A. calliptera* breeds from March to May in Lake Masoko^30^). There are also questions surrounding the efficacy of eDNA-based methods for informing on population level genetic processes, such as selection and mutation. Notably, the approach we used is similar to a pooled-sample sequencing approach (pool-seq), albeit with an unknown number of contributing individuals, and there may be potential to refine approaches for eDNA-based allele frequency analyses using those developed for pooled sequencing.^35^

## STAR★Methods

### Key resources table

**Table:** 

REAGENT or RESOURCE	SOURCE	IDENTIFIER
**Biological samples**		

Environmental DNA samples: Lake Masoko	This study	NA

**Critical commercial assays**		

DNeasy Blood and Tissue kit	Qiagen	Cat# 69504
OneStep PCR inhibitor removal kit	Zymo Research	Cat# D6030
NEBNext FFPE DNA Repair Mix, NEB	New England Biolabs	Cat# M6630S
NEBNext Ultra II End Repair/dA-Tailing Module	New England Biolabs	Cat# E7546S
AMPure XP SPRI reagent	Beckman Coulter	Cat# A63880
Qubit dsDNA Quantification Assay, Broad Range	Invitrogen-ThermoFisher	Cat# Q32850
NEBNext Quick Ligation Module	New England Biolabs	Cat# E6056S
Flongle pack for MinION	Oxford Nanopore Technologies	Cat# FLGIntSP
AmpliTaq Gold 360 Master Mix	Applied Biosystems - ThermoFisher	Cat# 4398881
QIAquick Gel Extraction Kit	Qiagen	Cat# 28704
Oligo Clean & Concentrator kit	Zymo Research	Cat# D4060
xGen UDI-UMI adapters	Integrated DNA Technologies	Cat# 10006914
PCR-free KAPA HyperPrep Kits	Roche	Cat# KK8503
NEBNext Library Quant Kit for Illumina	New England Biolabs	Cat# E7630L
NextSeq 500/550 High Output Kit v2.5 (75 cycles)	Illumina	Cat# 20024906

**Deposited data**		

Nanopore (ONT) sequence data – microbial eDNA	This study	Sequence Read Archive Bioproject: PRJNA985047
Illumina sequence data – cichlid amplicon eDNA	This study	Sequence Read Archive Bioproject: PRJNA985047
Analysis code and assets	This study	https://doi.org/10.5281/zenodo.10083982

**Oligonucleotides**		

Custom PCR oligonucleotides (Eurofins Genomics)	Ref.^14^ - See Table S2 for list	NA

**Software and algorithms**		

guppy	https://nanoporetech.com	v4.09
porechop	https://github.com/rrwick/Porechop	v0.2.4
KrakenUniq	https://github.com/fbreitwieser/krakenuniq	v0.7.3
vegan	https://github.com/vegandevs/vegan	v2.5-7
bwa	https://github.com/lh3/bwa	v0.7.17-r1188
SAMtools	https://github.com/samtools/samtools	v1.9
VCFtools	https://github.com/vcftools/vcftools	v0.1.16
gatk	https://github.com/broadinstitute/gatk	v4.3.0
BCFtools	https://github.com/samtools/bcftools	v1.8
R	https://www.r-project.org/	v4.2.3
plink	https://www.cog-genomics.org/plink/	v1.90b6.2
Admixture	https://dalexander.github.io/admixture/	1.3.0
pcaMethods	https://github.com/hredestig/pcaMethods	v1.90.0

**Other**		

Lake Massoko *A. calliptera* genotypes (VCF file)	Ref.^18^ – Raw sequence data in Sequence Read Archive Bioprojects: PRJEB1254, PRJEB10014 and PRJEB27804	NA
Reference genome (fAstCal1.2)	GenBank assembly	GCA_900246225.3
Microbial database	https://ccb.jhu.edu/software/kraken/	minikraken_20171019_8GB

### Resource availability

#### Lead contact

Further information and requests for resources should be directed to and will be fulfilled by the lead contact, Martin Genner (m.genner@bristol.ac.uk).

#### Materials availability

The study did not generate new unique reagents.

#### Data and code availability

• Sequence data have been deposited at the Sequence Read Archive and are publicly available as of the date of publication. Accession numbers are listed in the key resources table.
• All original code is publicly available as of the date of publication. DOIs are listed in the key resources table.
• Any additional information required to reanalyze the data reported in this paper is available from the lead contact upon request.

### Experimental model and study participant details

The study was based on analyses of environmental DNA samples and pre-existing genotyping data, it did not use experimental models or study human participants.

### Method details

#### Sampling eDNA in Lake Masoko

Water samples were collected in sterile containers using SCUBA from five depths in Lake Masoko (3, 7, 12, 18 and 22 m below the water surface) on the 3 September 2019 (Tables S1 and S3). In total we collected nine samples from 3 m depth, nine samples from 22 m depth, and four from each of 7 m, 12 m and 18 m depths. We used 25 of those 30 samples for the work reported here (Tables S1 and S3). All samples were collected from one location on the southeast of the lake (9.3362°S, 33.7566°E). A thermocline was present at the time of sampling, although temperatures were not recorded. Water samples were taken to the shore for processing. Two samples of bottled drinking water were processed as field controls. Research permission was issued by the Tanzania Commission for Science and Technology.

Each sample was filtered through a 0.22 μm Sterivex-GP polyethersulfone (PES) filter (Merck Millipore) using repeated loadings of a 60 ml syringe. We aimed to maximise the volume of water sampled in each filter, with the final volume reached when the filter became clogged. The total volume of water sampled in each filter was recorded. Water was expelled, and the outlet of the filter was sealed by melting the plastic. Environmental DNA within the filters was preserved by adding 0.37 ml of ATL buffer to the filter cartridge (Qiagen) using a 1 ml syringe, before a combistopper was used to seal the inlet of the Sterivex. Each sample was then individually placed in a WhirlPak bag, labelled, and stored in shade and away from heat sources. For long-term storage, filters were placed in -20°C freezer three days after collection. A detailed field collection protocol is at https://zenodo.org/record/4687985.

#### DNA extraction

Environmental DNA was extracted from Sterivex filters using a modified DNeasy Blood and Tissue kit (Qiagen) protocol (https://doi.org/10.5281/zenodo.4741283), in a dedicated trace DNA extraction laboratory. Each aliquot of extracted eDNA was passed through a OneStep PCR inhibitor removal column (Zymo Research). The quantity of eDNA in each sample was then quantified using spectrophotometry (NanoPhotometer N60-Touch, Implen).

#### ONT sequencing of microbial communities in eDNA

In total nine samples were used for ONT (Oxford Nanopore Technologies) sequencing of the bacterial community. These covered the sampled depth range, and included three samples from 3 m depth, three samples from 22 m depth, and one from each of 7 m, 12 m and 18 m depths (Table S1). Using a starting volume of 500 ng of DNA in 10 μl, we added 15μl of nuclease-free water, and used DNA repair (NEBNext FFPE DNA Repair Mix, NEB) and end-prep (NEBNext Ultra II End repair/dA-tailing Module, NEB) following the manufacturer’s protocol. The samples were then subject to a bead-clean up (30μl AMPure XP SPRI reagent, Beckman Coulter: 30μl of sample) using 75% fresh ethanol. After clean-up, 1μl was quantified using a Qubit 3.0 fluorometer with a Broad Range Assay (Invitrogen).

The DNA was then adaptor-ligated (NEBNext Quick Ligation Module, NEB) and cleaned again using AMPure XP beads (as above). An ONT buffer (either SFB or LFB) was used to wash beads and enrich fragments. Samples were stored in LoBind tubes (Eppendorf) and 1μl from each sample was quantified using the Qubit fluorometer, ensuring that they contained between 3 and 20 fmol. Samples were sequenced on Flongle flow cells for MinION (Oxford Nanopore Technologies) following the manufacturers protocol, ensuring a minimum active pore number of 60 prior to loading.

#### Illumina-based genotyping of fish eDNA

In total, 16 samples were used for analyses of fish SNP allele counts in the eDNA. These covered the sampled depth range, and included five samples from 3 m depth, five samples from 22 m depth, and two from each of 7 m, 12 m and 18 m depths (Table S3). We used a set of 100 pairs of PCR primers flanking regions containing SNPs, as identified by,^14^ to amplify target sequences (Table S2). The primers were assigned to 26 different groups according to annealing temperatures, and PCR reactions were performed in multiplex for each group. Three replicate PCRs were performed on each eDNA template with each primer group. Each PCR was conducted in a 10 μl volume comprising: 5 μl AmpliTaq Gold 360 Master Mix (Applied Biosystems); 0.5 μl forward primer from each group (5 μM); 0.5 μl reverse primer from each group (5 μM); 3 μl molecular-grade water; and 1 μl eDNA template. Thermocycling initially comprised a polymerase activation step at 95°C for 10 min. This was followed by 40 cycles of: denaturation at 95°C for 30 s, annealing (estimated annealing temperature plus 3°C–4°C) for 30 s, extension at 72°C for 60 s. The final extension was at 72°C for 10 min. The eDNA extractions, pre-PCR preparations, and post-PCR procedures were carried out in separate rooms. We tested the following controls for amplification: two field negatives (the bottled water filtered in the field), two laboratory extraction negatives (new Sterivex filters that underwent the extraction protocol), and one PCR negative consisting of molecular biology grade ultrapure water. There was no indication of an amplification product in any negative control visible on agarose gel.

PCR products from all 78 amplifications of each sample were pooled. These were loaded on agarose gels (2%), and amplicons excised and purified using the QIAquick Gel Extraction Kit (Qiagen) and the Oligo Clean & Concentrator kit (Zymo Research) using a modified version of the manufacturer’s protocol (https://doi.org/10.5281/zenodo.10083982). Illumina library preparation was conducted using xGen UDI-UMI adapters (IDT), ligated to the amplicons using the PCR-free KAPA HyperPrep Kit (Roche) following the manufacturer’s protocol. A total of 16 libraries using unique indexes were created. Libraries were then quantified individually using a NEBNext Library Quant Kit for Illumina (NEB) qPCR assay, standardized, and sequenced on an Illumina NextSeq 500 using v2.5 (75 bp paired-end reads) high output chemistry and 10% phiX spike-in.

### Quantification and statistical analysis

#### Bioinformatic analyses of microbial community ONT sequences

Base-calling and quality filtering of ONT data were performed using Guppy software v4.09 (https://nanoporetech.com). Sequencing adaptors were removed using porechop v0.2.4 (https://github.com/rrwick/Porechop). For assignment of reads to microbial taxa, we used KrakenUniq v0.7.3^36^ employing the minikraken_20171019_8GB microbial database (https://ccb.jhu.edu/software/kraken/). Reads were filtered to only include only those assigned to bacteria at the order level, which provided sufficient resolution for a broad interpretation of ecological preferences of constituent taxa in samples. To quantify community structure across the depth gradient we used a canonical correspondence analysis using the R package vegan v2.5-7,^37^ testing the association of the primary axes of variation with depth using the `anova.cca` function, with 10,000 permutations.

#### Bioinformatic analyses of genomic variants in eDNA

Demultiplexed Illumina sequencing reads were trimmed to remove adaptors using cutadapt v4.1.^38^ They were then mapped to an indexed *Astatotilapia calliptera* reference genome (fAstCal1.2; GCA_900246225.3) using bwa v0.7.17-r1188.^39^ Using SAMtools v1.9,^40^ SAM files were converted to BAM format using the view function, sorted using the sort function, read group information added using the addreplacerg function, and indexed using the index function. Mapping rates were determined using the flagstat function.

Ninety-eight primer pairs amplified genomic fragments located in the fAstCal1.2 genome based on a standard nucleotide BLAST (blastn; https://blast.ncbi.nlm.nih.gov/Blast.cgi) search (Table S2). We extracted variants from a VCF file of 648 Lake Masoko *A. calliptera* genomes (Sequence Read Archive bioprojects PRJEB1254, PRJEB10014 and PRJEB27804) aligned to the same ref.^18^ using a BED file containing the genomic coordinates of the 98 targeted fragments and VCFtools v0.1.16.^41^ This process yielded a set of 120 biallelic SNPs within the 98 loci. Across this set of 648 fish and across all chromosomes there was only weak linkage between SNPs (*r*^2^ = 0.071), but linkage within chromosomes was substantially greater (*r*^2^ = 0.672).

We counted the number of reads assigned to each allele at SNPs within the eDNA BAM files using the ASEReadCounter function in gatk v4.3.0.^42^ These allele count data were then manually curated into a combined file, including all samples, including total depth of the relevant alleles for each SNP (Table S4). We retained 71 focal SNPs that were represented in at least 75% of 16 eDNA samples.

We tested if reference allele frequencies of the 71 SNPs in eDNA were significantly associated with reference allele frequencies of SNPs in the fish for each of five depth strata (Table S3). We mapped our eDNA samples to the same depth strata used for fish collections [3 m eDNA collection = 0-5 m fish collection; 7 m eDNA collection = 5-10 m fish collection; 12 m eDNA collection = 10-15 m fish collection; 18 m eDNA collection = 15-20 m fish collection; 22 m eDNA collection = 20-25 m fish collection]. We calculated the average reference allele frequency of each SNP in samples from each depth within the eDNA data. We extracted the 71 focal SNPs present in the eDNA data from the VCF file of 648 aligned Lake Masoko *A. calliptera* individuals. Next, we separated this SNP-subsetted VCF file into five separate VCF files consisting of subsets of individuals sampled at different depths using the view function of BCFtools v1.8.^40^ We calculated the allele frequency among individuals from each depth range using the freq2 function in VCFtools v0.1.16.^41^ We compared allele frequencies within each depth stratum using the Pearson correlation coefficient (cor.test function) in R v4.2.3.^43^

We determined whether these 71 “eDNA SNPs” were able to resolve genetic structure over the Lake Masoko depth gradient, using genetic information of fish collected from known depths (530 of the 648 individuals). First, we pruned SNPs in linkage disequilibrium from the WGS data (3,107,901 of 3,881,258 variants removed) using plink v1.90b6.2,^44^ with a window size of 50 kb, a step size of 10 bp, and pruning sites above an r^2^ threshold of 0.1. We used Admixture 1.3.0^45^ to calculate ancestry proportions of individuals based on this set of 773,357 unlinked SNPs, assuming two ancestry components (K = 2). We compared these ancestry proportions to those of the eDNA samples calculated using the 71 focal SNPs.

We summarized variation across the 71 SNPs in the 16 eDNA samples (Table S3) with a Principal Component Analysis (PCA) using the R package pcaMethods v1.90.0.^46^ Finally, we compared shifts in allele frequencies from shallow (3 m eDNA; 0-5 m fish) to deep (22 m eDNA, 20-25 m fish) habitats between the eDNA and WGS datasets using the Pearson correlation coefficient (cor.test function in R).

## Supplemental information

Table S1. Samples used for nanopore sequencing of bacterial community composition, and outputs from the KrakenUniq assignment analysis

Table S2. Primers used for generation of SNP-containing amplicons Coordinates of two of the 100 primer pairs could not be located in the reference fAstCal1.2 genome, so were not included in data analyses, and are represented by NA in the “fAstCal1.2 position” and “Amplicon size” fields.

Table S3. Samples used for the eDNA analysis targeting *Astatotilapia calliptera* SNPs

Table S4. Numbers of reads with the reference and alternative alleles of 120 biallelic SNPs (across 98 loci) in 16 eDNA samples Codes refer to samples described in Table S3.
